# Macrominerals and Trace Element Requirements for Beef Cattle

**DOI:** 10.1371/journal.pone.0144464

**Published:** 2015-12-14

**Authors:** Luiz Fernando Costa e Silva, Sebastião de Campos Valadares Filho, Terry Eugene Engle, Polyana Pizzi Rotta, Marcos Inácio Marcondes, Flávia Adriane Sales Silva, Edilane Costa Martins, Arnaldo Taishi Tokunaga

**Affiliations:** 1 Universidade Federal de Viçosa, Animal Science Department, Viçosa, MG, Brazil; 2 Colorado State University, Animal Science Department, Fort Collins, CO, United States of America; Istituto Biologia e Biotecnologia Agraria IBBA, ITALY

## Abstract

Eighty-seven Nellore animals were utilized in this study to estimate net requirements for the maintenance and growth of beef cattle as well as the retention coefficients of 13 minerals: macrominerals (Ca, P, Mg, K, Na, and S) and trace elements (Cu, Fe, Mn, Se, Zn, Co, and Cr). The net requirements for maintenance and the true retention coefficient were estimated by using the regression between apparent retention and intake for each mineral. The net requirement for maintenance (μg/kg BW) and retention coefficients (%) were 163 and 85 for Cu, 2,097 and 53 for Fe, 32.3 and 24 for Mn, 3.72 and 48 for Se, 669 and 0.80 for Zn, 18.4 and 86 for Co, and 22.9 and 78 for Cr. The dietary requirements of macrominerals (g/kg DMI) were 5.12 for Ca, 2.38 for P, 0.96 for Mg, 2.40 for K, 0.79 for Na, and 1.47 for S. This is the first study using Nellore cattle to estimate mineral requirements; considering that Nellore cattle are the most common breed in Brazil and that Brazil is a major beef producer globally, this knowledge can help producers to improve animal performance by supplying the correct amount of minerals.

## Introduction

Minerals play a role in four types of functions in animals: structural, physiological, catalytic, and regulatory [1]. Thus, information regarding the mineral requirements for body maintenance and growth are essential for beef cattle to reach their maximum productive potential [2]. The beef cattle National Research Council (NRC) [3] suggested that at least 17 minerals are required by beef cattle; however, the requirements of the majority of minerals are suggested in grams or milligrams per kilogram of dry matter intake (**DMI**) without discrimination between body maintenance or growth status, and the absorption coefficient is not taken into account for trace elements and sulfur. Additionally, requirements for certain minerals are not listed because the available research results are inadequate for accurate determination [3]. Moreover, the available systems for the determination of nutrient requirements were developed using breeds that are uncommon in Brazil, such as Angus and Hereford cattle. Thus, it is necessary to undertake studies using Nellore cattle to determine nutrient requirements for this breed, which is the most utilized cattle breed in Brazil.

The ARC, AFRC, and NRC [3–5] consider mineral requirements based on mineral supplementation while disregarding mineral composition from feedstuffs. This, together with knowledge of the amount of each mineral that is absorbed and retained is important for meeting mineral requirements of cattle.

The ARC, AFRC, and NRC [3–5] consider the absorption coefficient of certain minerals because urinary excretion of certain minerals is negligible [3, 4]. However, the retention coefficient can directly represent the relationship between intake and retained minerals while also taking into account other possible mineral losses such as through the skin and urine [6]. Thus, the use of the true retention coefficient seems to be more accurate than the absorption coefficient when it comes to estimating mineral requirements.

We hypothesized that knowledge of the net requirements for maintenance and growth as well as the true retention coefficient will improve the precision of setting requirements for all minerals. Therefore, we developed two experiments to estimate macromineral (Ca, P, Mg, K, Na, and S) and trace element (Cu, Fe, Mn, Se, Zn, Co, and Cr) requirements for body maintenance and growth, as well as the true retention coefficient in Nellore beef cattle. The mineral balance method, based on the difference between mineral intake and mineral excretion (e.g. in feces and urine), was utilized to calculate mineral retention.

## Materials and Methods

Two experiments for the evaluation of mineral intake, retention, excretion, and dietary requirements were conducted at the Experimental Feedlot of the Animal Science Department in the Universidade Federal de Viçosa, Viçosa, Brazil. Animal care procedures throughout the study followed protocols approved by the Conselho Nacional de Controle de Experimentação Animal (CONCEA) guidelines (UFV number 10/2013).

### Animals and Treatments

#### Experiment I–bulls

Thirty-seven Nellore young bulls with an average initial body weight (**BW**) of 259 ± 25.1 kg and age of 14 ± 1.2 months were used. Five bulls were randomly designated as baseline reference group (**REF**) and were slaughtered at the beginning of the experiment, four bulls were fed at maintenance level (**MAI**), and the remaining 28 bulls had *ad libitum* access to feed throughout the experiment (**ADL**). One MAI bull and seven ADL bulls were slaughtered at four points throughout the experiment (42, 84, 126, and 168 d).

Sixteen animals from the ADL group were randomly selected and housed in a tie stall system to evaluate the digestibility of the diet. The remaining 12 bulls from the ADL group were housed in two collective stalls with concrete floor and individual electronic feeders (American Calan Inc., Northwood, NH) to measure individual feed intake. The total area of each stall was 50 m^2^, with 9 m^2^ under cover, and a collective concrete trough for water. Initially, all animals were weighed, identified with an ear tag, and treated for ectoparasites and endoparasites (Ivomec, Paulina, São Paulo, Brazil).

The diet was formulated according to the BR CORTE [6] for a daily BW gain of 1.30 kg. The diet consisted of 55% corn silage and 45% concentrate on a dry matter (**DM**) basis. The concentrate portion of the diet was formulated with ground corn, soybean meal, urea, ammonium sulfate, limestone, common salt, and a mineral mix (Table 1). Feed was supplied twice daily to the animals and adjusted to keep orts at 5 to 10% of the total supplied feed. Water was continuously available to the animals. The amount of supplied feed was recorded daily. The MAI animals were fed at 11 g DMI/kg BW. The feed ingredients in the concentrate were sampled directly from the storage silos each time the diets were manufactured. Feed samples were composited weekly, dried in a forced-ventilation oven (55°C) for 72 h, and ground through a 1 mm screen (Wiley mill; A. H. Thomas, Philadelphia, PA). At the end of each 7-d period, a composite sample of orts was made for each 42-d period proportionally to weight (DM basis) from each week. For the corn silage, the composite samples were made every 21 d.

**Table 1:** Proportions of feed in concentrate and the diet, and concentrate and diet composition calculated on a DM basis for experiment 1.

Ingredients	Concentrate	Diet
**Proportion (g/kg DM)**		
**Corn silage**	0.00	550
**Ground corn**	816	367
**Soybean meal**	137	62.0
**Urea**	18.0	8.00
**Ammonium sulfate**	2.00	1.00
**Salt**	10.0	5.00
**Limestone**	7.00	3.00
**Mineral mix** ^**1**^	10.0	4.00
**Chemical composition (g/kg DM)**		
**Dry matter (g/kg)**	876	555
**Crude protein**	195	123
**Neutral detergent fiber**	134	347
**Ca**	5.90	3.60
**P**	5.22	3.26
**Mg**	1.10	1.30
**K**	4.70	7.70
**Na**	3.00	1.60
**S**	1.30	1.01
**Cu (mg/kg DM)**	19.7	11.9
**Fe (mg/kg DM)**	164	388
**Mn (mg/kg DM)**	25.9	34.1
**Zn (mg/kg DM)**	76.0	45.2
**Co (mg/kg DM)**	1.90	1.50
**Cr (mg/kg DM)**	3.00	3.10

^1^266 g/kg calcium (calcium carbonate source); 147 g/kg phosphorus (dicalcium phosphate source); 7 g/kg magnesium; 3 g/kg potassium; 2 g/kg sodium (sodium chloride source); 7 g/kg sulfur (cobalt sulfate and zinc sulfate source); 1,191 mg/kg copper (copper chelate source); 5,070 mg/kg iron (iron sulfate source); 1,728 mg/kg manganese (manganese chelate source); 4,198 mg/kg zinc (zinc sulfate source); 136 mg/kg cobalt (cobalt sulfate source); 118 mg/kg chromium.

#### Experiment II–heifers and steers

Eighteen Nellore steers (150 ± 44.2 kg) and 32 Nellore heifers (180 ± 41.0 kg) were used in this experiment. Four animals per gender were used as REF animals and were slaughtered at the beginning of the experiment. Four animals from each gender were fed at MAI, and 10 steers and 24 heifers were assigned to the ADL group. The ADL and MAI heifers were further divided into four groups and assigned to the following dietary treatments: 1) Ca and P fed at requirements (**CaPR**) with a 50:50 roughage:concentrate (R:C) diet, 2) CaPR with a 70:30 R:C diet, 3) 43% Ca and 80% P requirements (**CaPL**) with a 50:50 R:C diet, and 4) CaPL with a 70:30 R:C diet. The nutrient requirements were considered to be those recognized by the BR CORTE [6]. The ADL and MAI steers were fed CaPR with a 50:50 R:C diet. Half of the ADL animals were slaughtered at d 50 and the other animals were slaughtered at d 100 of the feeding period. All of the MAI-fed cattle were slaughtered at d 100. The values of 43% Ca and 80% P were the lowest values that were achieved when this diet was provided to the animals.

The animals were housed in individual pens with concrete floors with a total area of 30 m^2^. This experiment was a completely randomized design with a 2 × 2 + 1 factorial arrangement of treatments which considered two roughage/concentrate ratios, two Ca and P levels, plus steers. The diet was formulated according to the BR CORTE system [6] for a daily BW gain of 0.3 kg. The diet consisted of fresh sugarcane and concentrate, which was formulated with ground corn, soybean meal, limestone, common salt, and a mineral mix (Table 2). The DM of sugarcane was assessed three times weekly to adjust the amount of urea and ammonium sulfate that was supplied to the animals. The urea/ammonium sulfate (**U/AS**) mixture was used to adjust the crude protein (**CP**) content of the diets to 124.0 g/kg DM (19.8 g of N/kg DM).

**Table 2:** Proportions of feed in each diet, and its composition calculated on a DM basis for experiment 2.

Items	70:30		50:50			Sugarcane
	CaPR	CaPL	CaPR		CaPL	
	**Proportion (g/kg dry matter)**					
**Sugarcane**	700	700	500		500	-
**Ground corn**	246	246	411		411	-
**Soybean meal**	45.1	45.1	75.2		75.2	-
**Dicalcium phosphate**	4.80	2.40	2.50		0.00	-
**Salt**	0.40	0.40	0.70		0.70	-
**Limestone**	3.10	0.00	5.30		2.20	-
**Mineral mix** ^**1**^	0.24	0.24	0.35		0.35	-
**Sand**	0.00	5.50	5.30		10.8	-
	**Chemical composition (g/kg dry matter)**					
**Dry matter (g/kg)**	488	488	599	599		322
**Crude protein**	162	162	163	162		34.0
**Neutral detergent fiber**	397	397	326	326		503
**Ca**	5.65	3.85	5.23	3.44		4.30
**P**	2.77	2.26	2.66	2.15		1.20
**Mg**	1.96	1.94	2.50	2.48		1.10
**K**	4.09	4.09	4.41	4.41		3.60
**Na**	0.43	0.42	0.57	0.56		0.20
** S**	1.29	1.26	1.38	1.34		1.10
** Cu (mg/kg DM)**	12.8	14.1	18.8	18.8		3.70
** Fe (mg/kg DM)**	329	341	275	287		422
** Mg (mg/kg DM)**	57.9	59.7	56.7	56.7		59.5
** Se (mg/kg DM)**	0.45	0.46	0.41	0.41		0.50
** Zn (mg/kg DM)**	44.8	47.3	60.3	59.8		19.7
** Co (mg/kg DM)**	1.64	1.52	1.50	1.37		1.60
** Cr (mg/kg DM)**	3.56	3.10	3.17	2.70		2.60

^1^29.2 g/kg calcium; 0.70 g/kg phosphorus; 2.11 g/kg magnesium; 0.89 g/kg potassium (potassium iodate source); 0.31 g/kg sodium (sodium chloride source); 63.5 g/kg sulfur (copper sulfate and zinc sulfate source); 3,296 mg/kg copper (copper sulfate source); 2,088 mg/kg iron (iron sulfate source); 4,673 mg/kg manganese (manganese sulfate source); 318 mg/kg of selenium (sodium selenite source); 7,817 mg/kg zinc (zinc sulfate source); 348 mg/kg cobalt (cobalt sulfate source); and 2.56 mg/kg chromium.

Feed was supplied twice daily to the animals and adjusted to keep orts at approximately 5 to 10% of the total supplied feed. Water was continuously available. The MAI animals were fed at 11 g/kg BW. The amount of supplied feed was recorded daily. The ingredients in the concentrate were sampled directly from the silos of the feed manufacturing facility each time they were mixed. Feed samples were obtained and composited as described for experiment I.

### Digestibility trial

For both experiments, a digestibility trial was conducted immediately before each slaughter period; total feces and urine were collected for three consecutive days from animals that were maintained in tie stalls [7]. At the end of each collection day, feces were weighed and homogenized, and a sample was collected. The samples were weighed, dried in a forced-ventilation oven (55°C) for 72 h, and ground through a 1 mm screen (Wiley mill; A. H. Thomas, Philadelphia, PA). One composite sample per animal, based on the DM weight for every collection day, was prepared.

Urine collection was performed via collecting funnels attached to the bulls and steers, while hoses carried the urine to tanks that were kept in polyethylene boxes with ice and 20% H_2_SO_4_ to reduce N loss. For heifers, a 2-way Foley catheter (No. 22, Rush Amber, Kamuting, Malaysia) with a 30-mL balloon was utilized to collect urine. A polyethylene tube was attached to the free end of the catheter, through which the urine flowed into a lidded plastic container that held 200 mL of 20% H_2_SO_4_. After each 24 h collection period, the total excreted weight of the urine was determined. The contents of all tanks were then homogenized. A 50-mL sample was obtained and was stored at −20°C for further laboratory analyses.

### Slaughter and Samplings

Before slaughter, all animals were fasted for 16 h to obtain the shrunk body weight (**SBW**). Animals were then slaughtered via captive bolt stunning followed by bleeding. After bleeding, the digesta was removed and discarded. The heart, lungs, liver, spleen, kidneys, the fat around the kidney, pelvis, and heart (KPH fat), diaphragm, mesentery, tails, trimmings, and washed gastrointestinal tracts were weighed. These values were added to the other parts of the body (i.e., carcasses, head, hide, limbs, and blood) to determine the empty body weight (**EBW**).

The rumen, reticulum, omasum, abomasum, small and large intestines, KPH fat, mesentery, liver, heart, kidneys, lung, tongue, spleen, diaphragm, esophagus, trachea, and reproductive tract were homogenized in an industrial cutter for 20 min. After removing the hide, the head and limbs were also ground in a bone crusher for 20 min. The hide was sampled in two parts to represent the shoulder, three parts to represent the dorsal line, two parts to represent the ventral line, two parts to represent the rear, one part to represent each foot, and one part to represent the head, which altogether represented the entire hide. A composite sample of non-carcass components was constructed in which blood, head, limbs, hide, organs, and viscera were sampled based on the relative proportions of each component after summing all weights of the components.

After slaughter, the carcasses of each animal were split into two half-carcasses which were chilled at 4°C for 18 h. After the 18 h-period, the half-carcasses were weighed again. The left half-carcass was completely separated into muscle, fat, and bone. Muscle and fat were ground together, and the bones were ground separately. A composite sample of the carcass was constructed by using relative individual proportions in the carcass. The non-carcass and carcass samples were lyophilized and ground in a ball mill for mineral analyses.

### Mineral Analyses

Corn silage (experiment I), sugarcane (experiment II), the concentrate, feces, urine, non-carcass, and carcass samples were analyzed for macrominerals (Ca, P, Mg, K, Na, and S) and trace minerals (Cu, Fe, Mn, Se, Zn, Co, and Cr). Calcium and Mg were determined by adding up lanthanum [8], and the readings were performed through atomic absorption spectrometry. For Na and K, concentrations were determined using flame emission spectrometry. Inductively coupled plasma-atomic emission spectroscopy with ultrasonic nebulization [9] was used for the determination of the following mineral concentrations: Co, Cr, Cu, Fe, Mn, Se, and Zn.

### Procedures Used to Calculate Mineral Requirements

#### Net requirements for maintenance and the true retention coefficient

The retained minerals in the body were determined for each mineral by subtracting the fecal and urinary mineral content from the mineral intake. The true retention coefficient and the net requirement of each mineral for maintenance were calculated based on regressions between the amounts of each macromineral (mg/kg EBW) and trace element mineral (μg/kg EBW) that was retained in the body and their intakes (Eq 1). Thus, a linear regression between the retained mineral content and its intake was performed by using the following regression model:

MR=β0+β1×MI+εi, (1)

where MR is the retained mineral in the body, MI is the mineral intake, β_0_ is the intercept and is considered to be the net requirement for the maintenance of each mineral, β_1_ is the slope and is considered to be the true retention coefficient, and ε_i_ is residual term.

#### Net requirements for growth

The amounts of each mineral in the body from each animal were estimated as a function of EBW according to the following model proposed by Brody [10]:

$$\text{Mi}=\beta 0\times {\text{EBW}}^{\beta 1}+\varepsilon \text{i},$$ (2)

where Mi = the macromineral (grams) or trace element (milligrams) content in the animal’s body, β_0_ and β_1_ = regression parameters from Eq 2 and ε_i_ is random error. Based on the model parameters presented above, the net growth requirements of each mineral per kilogram of empty body gain (**EBG**) were calculated by using the derivative of Eq 2 with respect to EBW as follows:

$$\frac{\text{d}}{\text{dEBW}}\text{f}\left(\text{EBW}\right)=\beta 0\times \beta 1\times {\text{EBW}}^{\beta 1-1}$$ (3)

where Y = net growth requirement of each mineral (macronutrient = g/kg EBG and trace element = mg/kg EBG).

#### Dietary requirements

After calculating the net requirements for maintenance and growth and the true retention coefficient for each mineral, the dietary requirements were calculated. The sum of the net requirements for maintenance and growth were divided by the true retention coefficient to estimate the dietary requirements.

### Running Analysis

To obtain the net requirements for maintenance and the true retention coefficient, the data for retained minerals were analyzed as in Eq 1 through the MIXED procedure of SAS (SAS version 9.3, Institute Inc., 2011, Cary, NC, USA). In instances where the parameters were different from zero, a Variance Component structure of the variance-covariance matrix was used to fit the equations. The NLIN procedure of SAS was used to fit the net requirements for growth as nonlinear models, and the Gauss method was selected for convergence. The parameter was considered to be different from zero if the P-value was less than 0.05.

## Results and Discussion

The descriptive statistics of the animals that were used in this study are shown in Table 3, while intake, excretion, and retention of each mineral are shown in Table 4. The study group was composed of animals with SBW between 121.0 and 591.5 kg; average daily gain (**ADG**) between 0 and 1.95 kg/d, and DMI between 1.18 and 11.1 kg/d.

**Table 3:** Descriptive statistics of data used in the present study (n = 87).

Items	Average	MSE^1^	Minimum	Maximum
Experiment I—Bulls				
**SBW** ^**2**^ **(kg)**	375	104	219	592
**EBW** ^**3**^ **(kg)**	344	99.2	192	549
**ADG** ^**4**^ **(kg/day)**	1.23	0.45	-0.01	1.95
**EBG** ^**5**^ **(kg/day)**	1.24	0.44	-0.01	1.87
**DMI** ^**6**^ **(kg/day)**	7.17	1.74	2.28	11.1
**TDN** ^**7**^ **(%)**	69.8	4.83	59.5	80.6
Experiment II—Heifers and steers				
**SBW (kg)**	207	45.8	121	300
**EBW (kg)**	182	41.4	104	266
**ADG (kg/day)**	0.35	0.24	-0.05	0.84
**EBG (kg/day)**	0.35	0.27	-0.02	0.83
**DMI (kg/day)**	3.50	1.43	1.18	6.24
**TDN (%)**	78.1	6.11	60.6	87.3

^1^MSE = mean square error

^2^SBW = shrunk body weight.

^3^EBW = empty body weight.

^4^ADG = average daily gain.

^5^EBG = empty body gain.

^6^DMI = dry matter intake.

^7^TDN = total digestible nutrient.

**Table 4:** Average mineral intake, excretion, and retention in bulls, steers, and heifers used in the experiments (n = 87).

Mineral	Intake	Feces	Urine	Retained
	g/day			
**Ca**	22.0	10.6	0.85	10.6
**P**	36.3	10.0	1.34	25.0
**Mg**	9.11	3.63	1.95	3.53
**K**	36.4	17.6	5.80	12.7
**Na**	6.91	4.36	2.39	0.26
**S**	5.23	3.01	1.82	0.40
	mg/day			
**Cu**	83.5	54.9	3.19	25.4
**Fe**	1,923	1,561	69.1	292
**Mn**	163	140	2.8	20.5
**Se**	2.01	1.63	0.13	0.25
**Zn**	283	242	11.4	29.4
**Co**	8.84	5.80	1.29	1.75
**Cr**	16.1	12.1	2.35	1.73

**Fig 1:**
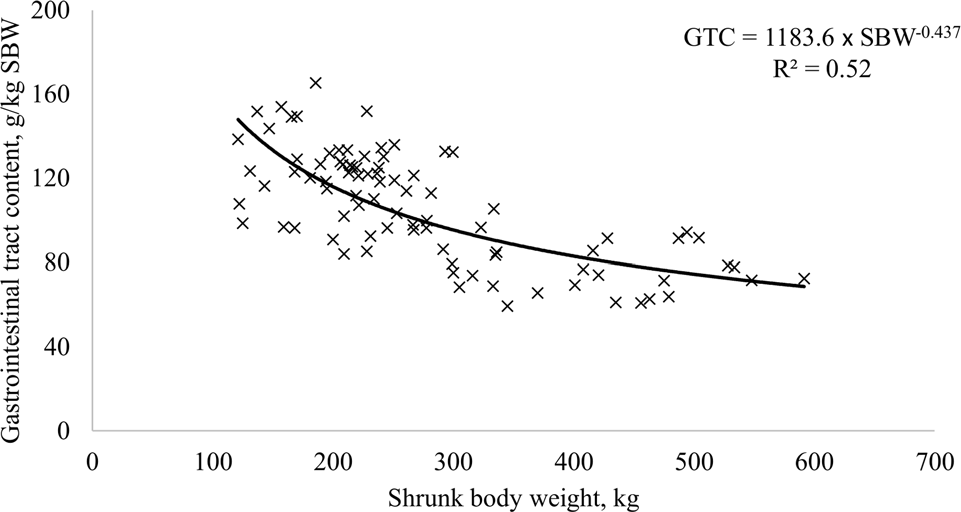
Relationship between gastrointestinal tract content (GCT, g/kg SBW) and shrunk body weight (SBW, kg).

The first step to meet dietary requirements is to estimate the empty body weight based on the shrunk body weight. In this context, the BR CORTE system [6] and the beef cattle NRC system [3] reported that the relationship between EBW and SBW is constant. However, our study indicated that this relationship might be different since the gastrointestinal content in the whole body decreased as the animal grew. Thus, we regressed the gastrointestinal tract content (**GTC**, g/kg SBW) on SBW (kg), from which GTC was calculated based on the difference between SBW and EBW (Fig 1). Furthermore, we assumed that the relationship between EBW and SBW is not constant and can be estimated by the following equations:

$$\text{GTC}=1,183.6\times {\text{SBW}}^{-0.437},$$ (4)

EBW=SBW−(0.001×GTC×SBW), (5)

where GTC is the gastrointestinal tract content (g/kg SBW), SBW is shrunk body weight (kg), and EBW is empty body weight (kg).

The BR CORTE system [6] and the beef cattle NRC system [3] suggest values of 0.891 and 0.895 for the relationship between EBW and BW, respectively. Young animals have a greater proportion of gastrointestinal tract contributing to BW. For example, a 100 kg animal would have 89.1 and 89.5 kg of EBW according to the BR CORTE system [6] and the beef cattle NRC system [3], respectively. The same animal would have 84.2 kg of EBW using the Eqs 4 and 5. However, a 400 kg animal would have 356.4, 358.0, and 365.5 kg EBW according to the BR CORTE system [6], the Beef cattle NRC system [3], and our calculations, respectively.

### Calcium

**Fig 2:**
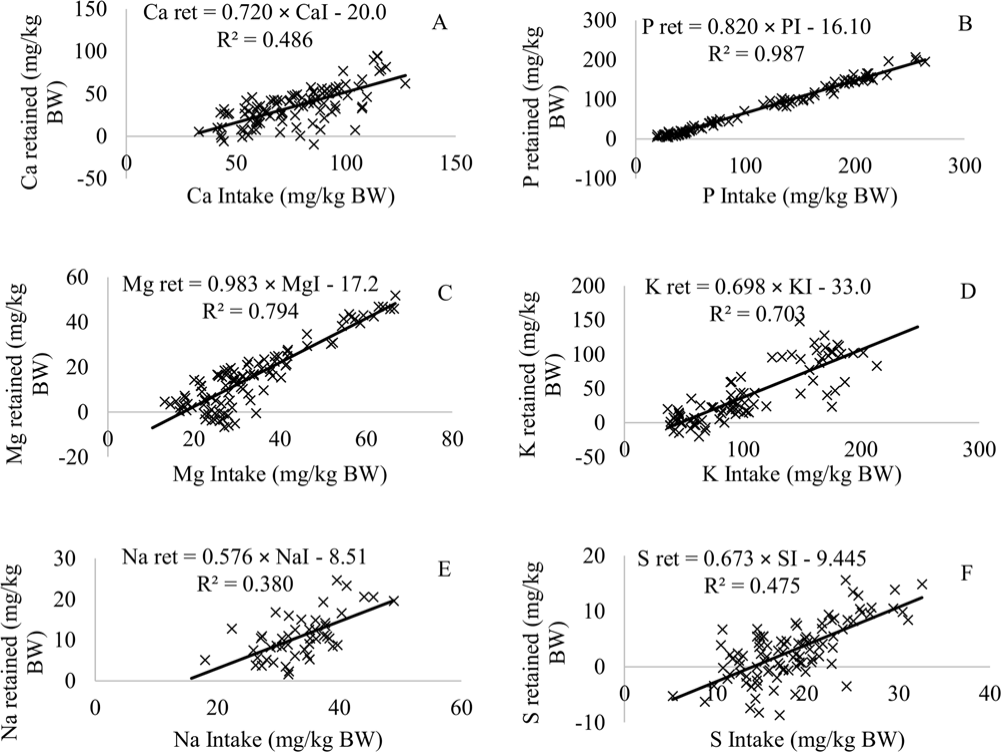
The net requirements for maintenance and the true retention coefficients of the macronutrient minerals for beef cattle. (**A**) Calcium, (**B**) Phosphorus, (**C**) Magnesium, (**D**) Potassium, (**E**) Sodium, (**F**) Sulfur.

Calcium is the most abundant mineral in the body [3]. Due to the importance of this mineral in the body, there is a need to estimate dietary Ca requirements. The net Ca requirement that was estimated in the present study for maintenance was 20.0 mg/kg BW (Fig 2A), which was similar to values reported in the beef cattle NRC system [3], the dairy cattle NRC system [11], and the ARC system [4] recommendations (Table 5). The true absorption coefficient of Ca observed the present study was similar to those suggested by the dairy cattle NRC system [11] and the AFRC system [5] (Table 5), although it was 72% greater (Fig 2A) than those reported in the beef cattle NRC system [3] and the BR CORTE system [6] (Table 5) However, the beef cattle NRC [3] and the BR CORTE [6] publications used the Ca absorption coefficients while discarding urinary losses. In our study, there was considerable mineral excretion in the urine for all of the assayed minerals (Table 4). Calcium excretion in the urine (3.8%) was lower than excretion of other minerals. Thus, we considered the retention coefficient to correctly estimate the mineral bioavailability. We therefore recommend 72% as the true retention coefficient for beef cattle.

**Table 5:** Comparison between the council’s recommendations and the values that were obtained in this study.

Items	Ca	P	Mg	K	Na	S	Cu	Fe	Mn	Se	Zn	Co	Cr
	Net requirement for maintenance												
	mg/kg body weight						μg/kg body weight						
**ARC (1965)**	16	-	-	-	-	-	-	-	-	-	-	-	-
**ARC (1980)**	-	12	3	-	6.8	-	7.1	-	-	-	45	-	-
**NRC (1996)**	15.4	16	-	-	-	-	-	-	-	-	12	-	-
**NRC (2001)**	15.4	-	3	38	15	-	-	-	2	-	45	-	-
**CSIRO (2007)**	-	-	-	-	-	-	4	-	-	-	55	-	-
**Valadares Filho et al. (2010)**	-	17.6	3.3	-	7	-	-	-	-	-	-	-	-
**Our result**	20	16.1	17.2	33	8.51	9.4	163	2,097	32.3	3.72	669	18.4	22.9
	Absorption or retention coefficient (%)												
**ARC (1980)**	-	-	29.4	100	-	-	6	-	-	-	30	-	-
**AFRC (1993)**	68	58	-	-	-	-	-	-	-	-	-	-	-
**NRC (1996)**	50	68	17	-	91	-	-	-	-	-	-	-	-
**NRC (2001)**	70	75	17	90	90	-	-	-	75	40–50	-	-	-
**CSIRO (2007)**	-	70	-	-	-	-	-	-	1	30	40	-	-
**Valadares Filho et al. (2010)**	55	-	-	-	19	-	-	-	-	-	-	-	-
**Our result**	72	82	98.3	69.8	57.6	67.3	84.7	52.7	23.6	48.7	80	85.6	78.4
	Dietary requirement												
	g/kg dry matter intake						mg/kg dry matter intake						
**ARC (1980)**	-	-	-	-	-	-	7.1	-	-	-	-	-	-
**NRC (1996)**	-	-	1	6	0.8	1.5	10	50	20	0.1	30	0.11	0.4
**NRC (2001)**	-	1	-	-	-	2	-	-	-	0.3	-	0.11	-
**CSIRO (2007)**	-	-	1.3	-	0.8	-	-	-	-	0.05	11.6	-	-
**Our result**	5.12	2.38	0.79	2.4	0.96	1.47	9.53	218	9.59	0.57	61	2.78	2.53

The net Ca requirement for growth can be estimated by the following equation: Ca = 0.21 × EBW^-0.94^, in which the amount of Ca is calculated as grams of Ca per kilogram of EBG. The negative exponent indicates that deposition of Ca is reduced when the animals grow and that younger animals require more Ca than older animals.

The dietary requirement for Ca was obtained from the sum of the net Ca requirements for maintenance and growth, and the subsequent division by the retention coefficient. Therefore, for a 300 kg beef cattle with 1.00 kg of ADG, the dietary Ca requirement would be 23.6 g/d (Table 6).

**Table 6:** Dietary mineral requirements of beef cattle when using the mineral balance method.

BW, kg	Minerals												
	Ca	P	Mg	K	Na	S	Cu	Fe	Mn	Se	Zn	Co	Cr
	The net requirement for growth												
	g/day						mg/day						
**100**	2.84	7.45	0.33	1.67	1.14	1.73	5.74	115	2.87	0.76	61.9	4.23	0.01
**200**	1.48	7.68	0.32	1.71	1.19	3.22	7.23	155	5.01	0.72	113	8.46	0.01
**300**	1.01	7.81	0.32	1.73	1.21	4.62	8.27	185	6.94	0.70	160	12.7	0.01
**400**	0.77	7.91	0.32	1.74	1.23	5.97	9.09	210	8.74	0.69	205	16.9	0.01
**500**	0.63	7.99	0.32	1.76	1.25	7.28	9.79	231	10.5	0.68	249	21.2	0.01
	Dietary Requirements												
	g/day						mg/day						
**100**	6.72	11.0	2.08	7.12	3.46	3.98	26.0	616	25.8	2.33	161	7.10	2.94
**200**	7.61	13.3	3.83	11.9	5.01	7.59	47.0	1091	48.6	3.02	308	14.2	5.85
**300**	9.73	15.4	5.59	16.7	6.54	11.1	67.4	1545	70.5	3.74	451	21.3	8.77
**400**	12.2	17.5	7.34	21.4	8.05	14.5	87.6	1990	91.8	4.47	591	28.4	11.7
**500**	14.7	19.5	9.09	26.2	9.55	17.8	108	2429	113	5.21	729	35.5	14.6

### Phosphorus

The net P requirement for maintenance estimated in the present study was 16.1 mg/kg BW (Fig 2B), which similar values recommended in the beef cattle NRC system [3] and ARC system [4] (Table 5). The true retention coefficient that was reported in this study (82%; Table 5) was greater than those presented elsewhere [3, 5, 9]. The absorption of P can vary based on the P content of forage and concentrate, as well as the mineral sources that are used to feed animals [5]. According to the beef cattle NRC system [3], in terms of availability, supplemental sources of phosphorus were ranked (greatest to least) as follows: dicalcium phosphate, defluorinated phosphate, and bone meal [12]. As we used dicalcium phosphate in this study as the main source of phosphorus, the higher true retention coefficient was expected.

The net P requirement for growth can be calculated by the following equation: P = 6.10 × EBW^0.04^, in which the amount of P in the body is calculated as grams of P per kilogram of EBG. The exponent was close to zero, which indicates that the net P requirement for growth is almost constant over time. A different response was reported in the BR CORTE system [6] where the net P requirement for growth decreases as the animal grows. The discrepancy between our results and values reported in the BR CORTE [6] publication may be due to the BR CORTE [6] using data from finishing animals in which bone and muscle deposition are low, and considering that phosphorus performs a structural function [3], the requirement for growth in this category of animal is also low.

The dietary P requirement for 300 kg beef cattle with 1.00 kg/d of ADG was calculated as 15.4 g/d (Table 6), which was close to those values recommended by the beef cattle NRC system [6] (15.91 g/d). Erickson et al. [13] reported that the dietary P requirement is lower than 1.60 g/kg DM. However, the dietary P requirement that was observed in this experiment was 2.38 g/kg DM (Table 5). The ARC system [4] also reported that the ratio between Ca and P for ruminant diets is important because both minerals function together in bone formation, and they recommend that the Ca:P ratio is between 1:1 and 2:1. However, the beef cattle NRC system [3] highlighted that the effect of the Ca:P ratio on ruminant performance has been overemphasized in the past [14, 15] and that dietary Ca:P ratios between 1:1 and 7:1 result in similar animal performance. The Ca:P ratio that was observed in the present study was 2.15:1.

### Magnesium

The estimated net Mg requirement for maintenance was 17.2 mg/kg (Fig 2C). This value is greater than those suggested by the ARC system [4], the dairy cattle NRC system [3], and the BR CORTE system [6] (Table 5). According to the ARC system [4], the urinary endogenous loss of Mg has been ignored. However, our results (Table 4) show that the urinary excretion of Mg represented 21.4% of the total consumed Mg. Thus, we believe that the urinary excretion is important and it should be considered. Based on this data, the true retention coefficient was calculated as 98.3% (Fig 2C). The ARC system [4] reported an overall mean value of 29.4%, but for the calculation of allowances which provide a margin of safety, the lower value of 17.0% was recommended and used by the beef cattle NRC system [3] and the dairy cattle NRC system [11] as the absorption coefficient of Mg. The major difference observed between our results and requirements suggested by the ARC system [4] could be due to the amount of Mg that is released in urine and the breed of the animals that were used in the experiments.

The net Mg requirement for growth can be estimated by the following equation: Mg = 0.35 × EBW^-0.02^, in which the exponent is close to zero. This indicates that the net requirement for growth does not vary considerably when the animal grows. When the dietary requirement was expressed as grams per kilogram of DMI, the value that was presented in this study was 0.79, while those suggested by the beef cattle NRC system [3] and the CSIRO system [16] were 1.00 and 1.30 mg/kg DMI, respectively.

### Potassium

The ARC system [4] separated endogenous losses such as feces (2.6 g/kg DMI), urine (37.5 mg/kg BW), saliva (0.7 g/100 kg BW), and skin (1.1 g/d) by reporting different estimates for each loss for K. Therefore, the net K requirements for maintenance can be achieved by considering the sum of each loss. However, the data used by the ARC system [4] were from one study [17] that utilized nine heifers in a 3 × 3 Latin square to evaluate mineral balance. Thus, when aiming to standardize this estimate, we estimated the net K requirements for maintenance to be 33.0 mg/kg BW/d while accounting for all losses (Fig 2D). When considering a 300-kg animal with a DMI of 7.5 kg/d, we estimated the net K requirement for maintenance using the ARC system [4] to be 33.95 g/d. This value was greater than those observed in this study (9.9 g/d) and those proposed by the dairy cattle NRC system [11] at 11.4 g/d (38 mg/kg BW). These differences can be explained by the low number of observations that generated the estimates for the ARC system [4], while in this study, we utilized 87 observations, which provided a greater precision of the estimate.

Ward [18] reported that K is absorbed from the rumen and omasum as well as from the intestine. The ARC system [4] used the absorption coefficient to convert net to dietary requirements and assumed 100% as the K absorption coefficient. However, in this study, the retention coefficient was calculated as 69.8% (Fig 2D; Table 5). Ward [18] also indicated that urine is the major route of K excretion, and body reserves of K are minimal. Our results suggest that this cannot be accurate, as we have shown that the urine excretion represents only 16% of the animal's intake, while the fecal excretion and the amount that is retained by the cattle are 49 and 35%, respectively. The dairy cattle NRC system [11] reports a true absorption coefficient of 90%. Therefore, we recommend the use of 69.8% as the true retention coefficient for K.

The net K requirement for growth can be estimated by the following equation: K = 1.43 × EBW^0.03^, in which the exponent is close to zero indicating that the net requirement for growth does not vary considerably when the animal grows. However, the BR CORTE system [6] found that the net K requirement for growth increases when the animal grows. For a 300-kg beef cattle with 1.00 kg/d of ADG, the dietary K requirement would be 46.5 g/d (Table 6). This value is greater than the value of 34.4 g/d recommended for the same animal category by the BR CORTE system [6].

### Sodium

The ARC system [4] suggests that dietary Na can be freely and completely absorbed by cattle, and that the concept of strict endogenous fecal loss does not apply to Na. However, our data shows that there was a considerable amount of Na lost in the feces and urine (Table 4; 63.1 and 34.6% of the total consumed amount, respectively). Based on this data, the net Na requirement for maintenance was 8.51 mg/kg BW (Fig 2E), which was close to those values recommended by the ARC system [4] and the BR CORTE system [6] (Table 5); however, it was lower than those suggested by the dairy cattle NRC system [11](15 mg/kg BW). The beef cattle NRC system [3] and the dairy cattle NRC system [11] recommend 91 and 90% as the absorption coefficient for Na, respectively. However, Bankir et al. [19] reported that vasopressin increased Na reabsorption, and between 65 to 80% of the filtrate is reabsorbed. In the present study, we estimated the true retention coefficient for Na to be 57.6%, which was greater than the value proposed by the BR CORTE system [6], which was 19%.

The net Na requirement for growth can be estimated by the following equation: Na = 0.89 × EBW^0.05^, in which the exponent approaches close to zero indicating that the net requirement for growth does not vary considerably when the animal grows. When the dietary requirement is represented as grams per kilogram of DMI, the value that was presented in this study was 0.96, which was close to those suggested by the beef cattle NRC system [3] and the CSIRO system [16], which were both 0.80 mg/kg DMI.

### Sulfur

Despite the fact that the beef cattle NRC system [3] and the dairy cattle NRC system [11] reported that S requirements of beef cattle were not well defined, they recommended that dietary S requirements should be between 1.5 and 2.0 g/kg DMI. In fact, the net S requirement for beef cattle maintenance and the true retention coefficient have not been evaluated. In this study, maintenance requirements and the retention coefficients were 9.4 mg/kg BW and 67%, respectively. The net S requirement for growth was estimated by the following equation: S = 0.03 × EBW^0.89^. The net S requirement for growth had different characteristics than those of other macronutrient minerals, because most of the macronutrient mineral requirements decline or do not change when the animal grows. For a 300 kg beef cattle with 1.00 kg of ADG, the dietary S requirement would be 10.5 g/d (Table 6). When expressed as gram per kilogram of DMI, the dietary S requirement is 1.47 g/kg DMI, which was close to the recommendations of the beef cattle NRC system [3] but lower than that of the dairy cattle NRC system [11]. This difference might be because data from the dairy cattle NRC system [11] was provided by only one study [20] that used mid-lactation dairy cows producing 30 to 37 kg milk/d.

### Copper

**Fig 3:**
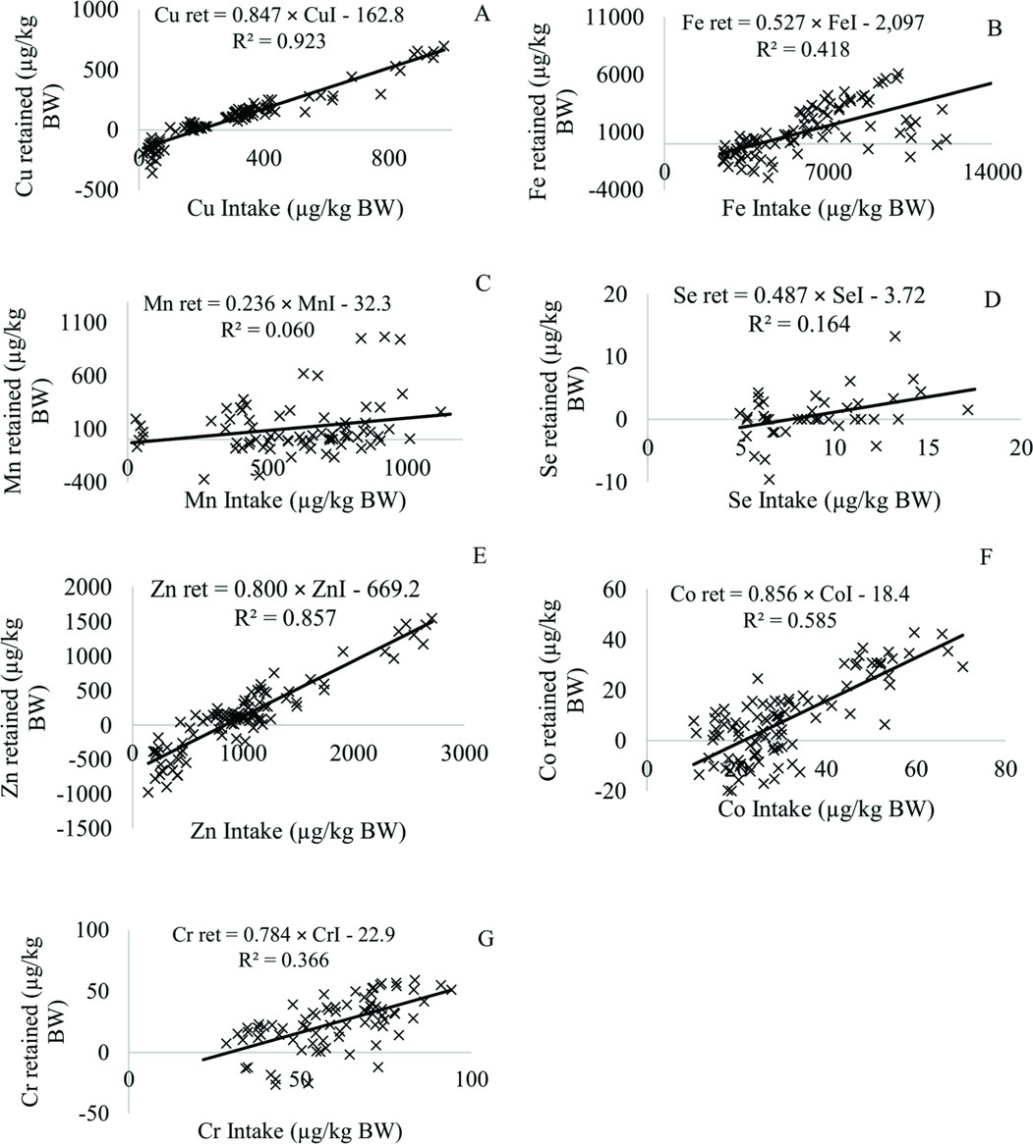
The net requirement for maintenance and the true retention coefficients of trace element minerals for beef cattle. (**A**) Copper, (**B**) Iron, (**C**) Manganese, (**D**) Selenium, (**E**): Zinc, (**F**) Cobalt, (**G**) Chromium.

The ARC system [4] reported that the net Cu requirement for maintenance was 7.1 μg/kg BW; however, this value was generated from an equation with variables such as Cu intake, hepatic Cu loss, and change in BW. Each of these components in the equation has certain assumptions. Also, two studies involving cattle yielded estimates of 1.8 and 0.8 μg/kg BW, respectively [21, 22]. The CSIRO system [16] adopted 4.0 μg/kg BW as the net Cu requirement for maintenance based on a study developed by Suttle [23]. On the contrary, the net Cu requirement for maintenance in this study was 163 μg/kg BW, which is greater than those reported by the ARC system [4] and the CSIRO system [16]. Also, the ARC system [4] estimated the Cu absorption coefficient as 6% when using the hepatic Cu retention technique. In this study, the true retention coefficient when using the mineral balance technique was 84.7% (Fig 3A), thus showing a value that is greater than those proposed by the ARC system [4]. However, the ARC system [4] reported that the efficiency of absorption and the hepatic retention of copper have not been detected in cattle, and that the recommendation of this council was provided based on data using sheep. Thus, we recommend the use of 84.7% as the true retention coefficient for beef cattle. Also, the net Cu requirement for growth can be estimated by the following equation: Cu = 1.25 × EBW^0.33^ in which Cu had a similar response to that observed for S, since the Cu requirement for growth increases as an animal grows.

Mullis et al. [24] estimated Cu requirements of Angus and Simmental heifers to be 7 mg/kg DMI. However, these authors did not consider the amount of Cu in the diet. Therefore, the Cu requirement might be lower than the value that was suggested in this study. A dietary Cu requirement of 9.53 mg/kg DMI should be adequate (Table 5).

### Iron

Thomas [25] reported that insufficient dietary Fe will reduce body stores as well as plasma Fe and blood hemoglobin concentrations. However, no studies have attempted to estimate the net Fe requirement for maintenance and the true retention coefficient. The net Fe requirement for maintenance that was estimated in this study was 2,097 μg/kg BW, while the true retention coefficient was 52.7% (Fig 3B). The net Fe requirement for growth can be estimated by the following equation: Fe = 15.5 × EBW^0.43^.

Bremner and Dalgarno [26] evaluated Fe requirements in calves and recommended that a dietary intake of 40 mg of soluble Fe/kg DMI is enough to prevent the development of anemia. Bernier et al. [27] recommended an additional Fe supplementation between 30 and 50 mg/kg DMI to avoid anemia. The large difference between the beef cattle NRC system [3] recommendation and our data (50 *vs*. 218 mg/kg DMI; Table 5) may be due to differences in basal diet Fe concentrations, physiological status of the animals, and breed differences across studies.

### Manganese

The body of a normal 70 kg animal is estimated to contain a total of 10 to 20 mg Mn [28]. The amount of Mn in the body is distributed widely throughout the tissues and fluids and may vary with age, species, organs, and in relation to other dietary trace elements. Schroeder et al. [29] assumed that 20 to 25 mg Mn/kg DMI is necessary for an animal to have optimum skeletal development. However, no study has evaluated the net Mn requirement for maintenance. The net Mn requirement for maintenance in this study was 32.3 μg/kg BW and the true retention coefficient was estimated to be 23.6% (Fig 3C). The dairy cattle NRC system [11] suggested that the net Mn requirement for maintenance and the true absorption coefficient is 2.00 μg/kg BW and 75%, respectively. Although, some authors [30–32] have reported that approximately 1 to 4% of dietary Mn is absorbed, irrespective of dietary concentration. Hurley and Keen [33] reported that several factors, including a high concentration of Ca, P, and Fe in the diet may decrease Mn absorption.

The net Mn requirement for growth can be estimated by the following equation: Mn = 0.07 × EBW^0.80^. Bentley and Phillips [34] concluded that 10 mg/kg DMI can meet the Mn requirements for growth in young heifers. This value was reported by the BR CORTE system [6] as the Mn requirement for growth. Hartmans [35] fed cows for 2.5 to 3.5 years with diets containing 16 to 21 mg of Mn/kg diet DM, and did not report any signs of Mn deficiency or improvements in animal performance when supplementing Mn. In this study, the dietary Mn requirement was estimated to be 9.59 mg/kg DMI, which was lower than the recommendation of 20 mg/kg DMI by the beef cattle in the NRC system [3].

### Selenium

The Se concentration in the body is dependent upon the amount and chemical form of Se in the diet, as well as the type of tissue that is evaluated. High concentrations can occur in the liver and kidneys, but the largest amount of Se is sequestered by muscles [36]. There are no published experiments that have evaluated the net Se requirement for maintenance or the retention coefficient for beef cattle. The net Se requirement for maintenance was estimated to be 3.72 μg/kg BW in the current study. Wright and Bell [37] evaluated the absorption coefficient in sheep and swine and found that 35% of ingested isotopic Se was absorbed in sheep. The value in this study (48.7%; Fig 3D) is greater than values reported by Wright and Bell [37] and the CSIRO system [16]; however, it was close to the dairy cattle NRC system [11] recommendation (Table 5). The net Se requirement for growth can be estimated by the following equation: Se = 1.07 × EBW^-0.07^.

Oh et al. [38] fed lambs with milk and increasing Se concentrations from 0.01 to 0.05 mg/kg DMI, and observed an increase in glutathione peroxidase activity, but the maximal enzyme activity was not obtained until the diet provided at least 0.1 mg Se/kg DMI. However, these authors did not consider the amount of Se provided by milk. The dietary Se requirement was estimated to be 0.57 mg/kg DMI, which is greater than those values observed by Oh et al. [38] and reported in the CSIRO system [16], though they were close to those suggested by the dairy cattle NRC system [11] (Table 5).

### Zinc

Some researchers [39–41] have reported that the Zn concentrations in plants and animals are often comparable to those of Fe and that are usually greater than those of most other trace elements [42]. The beef cattle NRC system [3] used an average of several studies [43–45] to estimate the endogenous urinary Zn loss of 12 μg/kg BW (ranging from 4 to 19 μg/kg BW). Weigand and Kirchgessner [46] evaluated the Zn requirements of lactating dairy cows and estimated that the net Zn requirement for maintenance to be 53 μg/kg BW. The dairy cattle NRC system [11] and ARC system [4] estimated the net Zn requirement for maintenance to be 55 μg/kg BW, while the CSIRO system [16] estimate the net Zn requirement to be 45 μg/kg BW. These values are lower than those that were estimated in the present study (669 μg/kg BW; Fig 3E). The ARC system [4] also used an absorption coefficient for Zn of 30% for young growing ruminants, and a value of 20% for mature animals based on data from several studies [44–48]. The CSIRO system [16] adopted a true absorption coefficient of 60% for pre-ruminant calves and 40% for older animals with a functional rumen [49]. Our estimate, based on the retention coefficient, was calculated to be 80%.

The net Zn requirement for growth can be estimated by the following equation: Zn = 1.16 × EBW^0.86^. The ARC system [4] suggests that 16 to 31 mg Zn/kg BW may be incorporated into body tissue for each kilogram of body weight gained. The values for dietary Zn requirements in this study were greater than the values reported in the beef cattle NRC system [3] recommendations (Table 5). However, the data that provided the beef cattle NRC system [3] with estimates were from studies [50, 51] that evaluated the growth response to additional Zn supplementation where the Zn content in the basal diet was unknown in certain studies.

### Cobalt

Smith [52] reported that the efficiency in which animals obtain vitamin B_12_ from dietary Co is very low. Some studies [53, 54] verified that 84 to 98% of the dietary Co appeared in the feces within 5 to 14 days. However, the net Co requirement for maintenance was 18.4 μg/kg BW, while the true retention coefficient was 85.6% (Fig 3F), thus showing a different response from the previous studies. The net Co requirement for growth can be estimated by the following equation: Co = 0.04 × EBW^1.00^. The net Co requirement for growth increases proportionally to increases in animal body weight.

Smith [52] also suggested that the dietary Co requirement was 0.11 mg/kg DMI. This value was adopted by the beef cattle NRC system [3] and the dairy cattle NRC system [11], but they did not consider the absorption coefficient and feed composition. In the present study, the average dietary Co requirement was 2.78 mg/kg DMI (Table 5).

### Chromium

No study was specifically conducted to evaluate the net Cr requirements for maintenance and growth or the true retention coefficient. However, other studies [55, 56] evaluated Cr supplementation in calves and suggested that the addition of Cr at 0.4 mg/kg DMI increased the glucose clearance rate. Bernhard et al. [57] evaluated the effects of Cr supplementation on the performance of steers and observed a difference in ADG between steers without Cr supplementation and those that were supplemented with 0.3 mg of Cr per kilogram of DMI. In our study, the net Cr requirement for maintenance was 22.9 μg/kg BW, while the true retention coefficient was calculated as 78.4% (Fig 3G). The net Cr requirement for growth can be estimated by the following equation: Cr = 0.23 × EBW^0.61^, in which the amount of Cr is calculated as milligram of Cr per kilogram of EBG. For a 300 kg beef cattle with 1.00 kg of ADG, the dietary Cr requirement is 18.6 mg/d (Table 6). This value represents 2.53 mg/kg DMI, which is greater than published data (0.2–0.4 mg/kg DMI; Table 5).

## Conclusions

The use of the true retention coefficient improves the estimates of dietary requirements for Na, K, Mg, and S due to the considerable excretion of these minerals in urine. Also, the use of the net requirements for maintenance and growth and the true retention coefficient for the majority of the trace minerals resulted in different estimates than those obtained from the literature. This study provides information about mineral nutrition of Nellore cattle and would be useful in dietary formulations in countries that use this breed, such as Brazil.

## Supporting Information

S1 Spreadsheet The spreadsheet included as supporting information can be used to meet mineral requirements for maintenance and growth such as dietary requirements using different body weights and average daily gain. (XLSX)

S2 Spreadsheet Data showing how mineral requirements for maintenance and growth were achieved. (XLS)
